# Classification and specific primer design for accurate detection of SARS-CoV-2 using deep learning

**DOI:** 10.1038/s41598-020-80363-5

**Published:** 2021-01-13

**Authors:** Alejandro Lopez-Rincon, Alberto Tonda, Lucero Mendoza-Maldonado, Daphne G. J. C. Mulders, Richard Molenkamp, Carmina A. Perez-Romero, Eric Claassen, Johan Garssen, Aletta D. Kraneveld

**Affiliations:** 1 Division of Pharmacology, Utrecht Institute for Pharmaceutical Sciences, Faculty of Science, Utrecht University, Universiteitsweg 99, 3584 CG Utrecht, The Netherlands; 2 UMR 518 MIA-Paris, INRAE, c/o 113 rue Nationale, 75103 Paris, France; 3 Hospital Civil de Guadalajara “Dr. Juan I. Menchaca”, Salvador Quevedo y Zubieta 750, Independencia Oriente, C.P. 44340 Guadalajara, Jalisco México; 4 Department of Viroscience, Erasmus Medical Center, Rotterdam, The Netherlands; 5 Departamento de Investigación, Universidad Central de Queretaro (UNICEQ), Av. 5 de Febrero 1602, San Pablo, 76130 Santiago de Querétaro, QRO Mexico; 6 Athena Institute, Vrije Universiteit, De Boelelaan 1085, 1081 HV Amsterdam, The Netherlands; 7 Department Immunology, Danone Nutricia research, Uppsalalaan 12, 3584 CT Utrecht, The Netherlands

**Keywords:** Computer science, Viral infection, Classification and taxonomy

## Abstract

In this paper, deep learning is coupled with explainable artificial intelligence techniques for the discovery of representative genomic sequences in SARS-CoV-2. A convolutional neural network classifier is first trained on 553 sequences from the National Genomics Data Center repository, separating the genome of different virus strains from the Coronavirus family with 98.73% accuracy. The network’s behavior is then analyzed, to discover sequences used by the model to identify SARS-CoV-2, ultimately uncovering sequences exclusive to it. The discovered sequences are validated on samples from the National Center for Biotechnology Information and Global Initiative on Sharing All Influenza Data repositories, and are proven to be able to separate SARS-CoV-2 from different virus strains with near-perfect accuracy. Next, one of the sequences is selected to generate a primer set, and tested against other state-of-the-art primer sets, obtaining competitive results. Finally, the primer is synthesized and tested on patient samples (n = 6 previously tested positive), delivering a sensitivity similar to routine diagnostic methods, and 100% specificity. The proposed methodology has a substantial added value over existing methods, as it is able to both automatically identify promising primer sets for a virus from a limited amount of data, and deliver effective results in a minimal amount of time. Considering the possibility of future pandemics, these characteristics are invaluable to promptly create specific detection methods for diagnostics.

## Introduction

The Coronaviridae family presents a positive sense, single-strand RNA genome. These viruses have been identified in avian and mammal hosts, including humans. Coronaviruses have genomes from 26.4 kilo base-pairs (kbps) to 31.7 kbps, with G + C contents varying from 32 to 43%; human-infecting coronaviruses belonging to this family include SARS-CoV, MERS-CoV, HCoV-OC43, HCoV-229E, HCoV-NL63 and HCoV-HKU1^1^. In December 2019, SARS-CoV-2, a novel, human-infecting Coronavirus was identified in Wuhan, China, using Next Generation Sequencing (NGS)^2^. As of the 12th August of 2020, the new SARS-CoV-2 has 20,162,474 confirmed cases across almost all countries, with 3,641,603 cases in the European region^3^. In addition, SARS-CoV-2 has an estimated mortality rate of 3–4%, and it is spreading faster than SARS-CoV and MERS-CoV^4^.

As a typical RNA virus, new mutations appear every replication cycle of Coronavirus, and its average evolutionary rate is roughly 10^–4^ nucleotide substitutions per site each year^2^. In the specific case of SARS-CoV-2, RT-qPCR testing using primers in ORF1ab and N genes have been used to identified the infection in humans^5^. This method has come into question; Yang et al. in a study from 866 respiratory specimens showed that for 0–7 days after onset of illness, the sputum samples had a negative rate of 11.1% in severe and 17.8% in mild cases, follow by 26.7% and 27.0% in nasal swabs and finally 40% and 38.7% for throat swabs^6^. Zhao et al. reports that 35.2% of 173 patients did not show positive in RT-PCR test^7^, which has been further explored by Arevalo et al.^8^ and Woloshin et al.^9^. These problems could be the result of the variation of viral RNA sequences within virus species, and the viral load in different anatomic sites^10^. It has been noted that, population mutation frequency of site 8872 located in ORF1ab gene and site 28,144 located in ORF8 gene gradually increased from 0 to 29% as the epidemic progressed^11^. Apart from the false negative test problems, SARS-CoV-2 assays can yield a small portion of false positives through nonspecific detection of other Coronaviruses, as the virus is closely related to other Coronavirus organisms^12^. In addition, SARS-CoV-2 may be present with other respiratory infections, hindering its identification^13,14^.

Thus, it is fundamental to improve existing diagnostic tools to contain the spread. For example, diagnostic tools combining computed tomography (CT) scans with deep learning have been proposed, achieving an improved detection accuracy of 82.9%^15^. Another solution being used for studying SARS-CoV-2, is sequencing of the viral complementary DNA (cDNA). For example, we can use this sequencing data with cDNA, resulting from the PCR of the original viral RNA; e.g. Real-Time PCR amplicons to identify the SARS-CoV-2^16^.

Classification using viral sequencing techniques is mainly based on alignment methods such as FASTA^17^ and BLAST^18^. These methods rely on the assumption that cDNA sequences share common features, and their order prevails among different sequences^19,20^. However, these methods suffer from the necessity of needing base sequences for the detection^21^. Nevertheless, it is necessary to develop innovative improved diagnostic tools that target the genome to improve the identification of pathogenic variants, as sometimes several tests are needed to have an accurate diagnosis. Therefore, as an alternative, deep learning methods have been suggested for classification of DNA sequences. The advantage of these methods are that they do not need pre-selected features to identify or classify DNA sequences. Deep Learning has been efficiently used for classification of DNA sequences, using one-hot label encoding and Convolutional Neural Networks (CNN)^22,23^, albeit the examples in literature are featuring DNA sequences of length up to 500 bps, only.

In particular, for the case of viruses, NGS genomic samples might not be identified by BLAST, as there are no reference sequences valid for all genomes, as viruses have high mutation frequency^24^. Alternative solutions based on deep learning have been proposed to classify viruses, by dividing sequences into pieces of fixed length, ranging from 300 bps^24^ to 3000 bps^25^. However, this approach has the negative effect of potentially ignoring part of the information contained in the input sequence, that is disregarded if it cannot completely fill a piece of fixed size. The global impact of SARS-CoV-2 prompted researchers to apply effective alignment-free methods to the classification of the virus: for example, in^26^ the authors propose the use of Machine Learning Digital Signal Processing for separating the virus from similar strains, with remarkable accuracy. Nevertheless, there is no human-readable information that can be extracted from their black-box procedure, so the biological insight provided by their approach is limited. In order to offer further understanding to experts, techniques from the field of explainable AI (XAI)^27,28^ could be potentially effective; and it is interesting to remark that similar consideration specifically for the medical domain have already appeared in literature^29^.

Given the impact of the world-wide outbreak, international efforts have been made to simplify the access to viral genomic data and metadata through international repositories, such as the National Genomics Data Center (NGDC) repository^11^, the National Center for Biotechnology Information (NCBI) repository^30^ and the Global Initiative on Sharing All Influenza Data (GISAID) repository^31^, expecting that the ease of access to information would make it possible to develop medical countermeasures to control the disease worldwide, as it happened in similar cases earlier^32–34^. Thus, taking advantage of the available information of international resources without any political and/or economic borders, we propose an innovative system based on viral gene sequencing.

**Figure 1:**
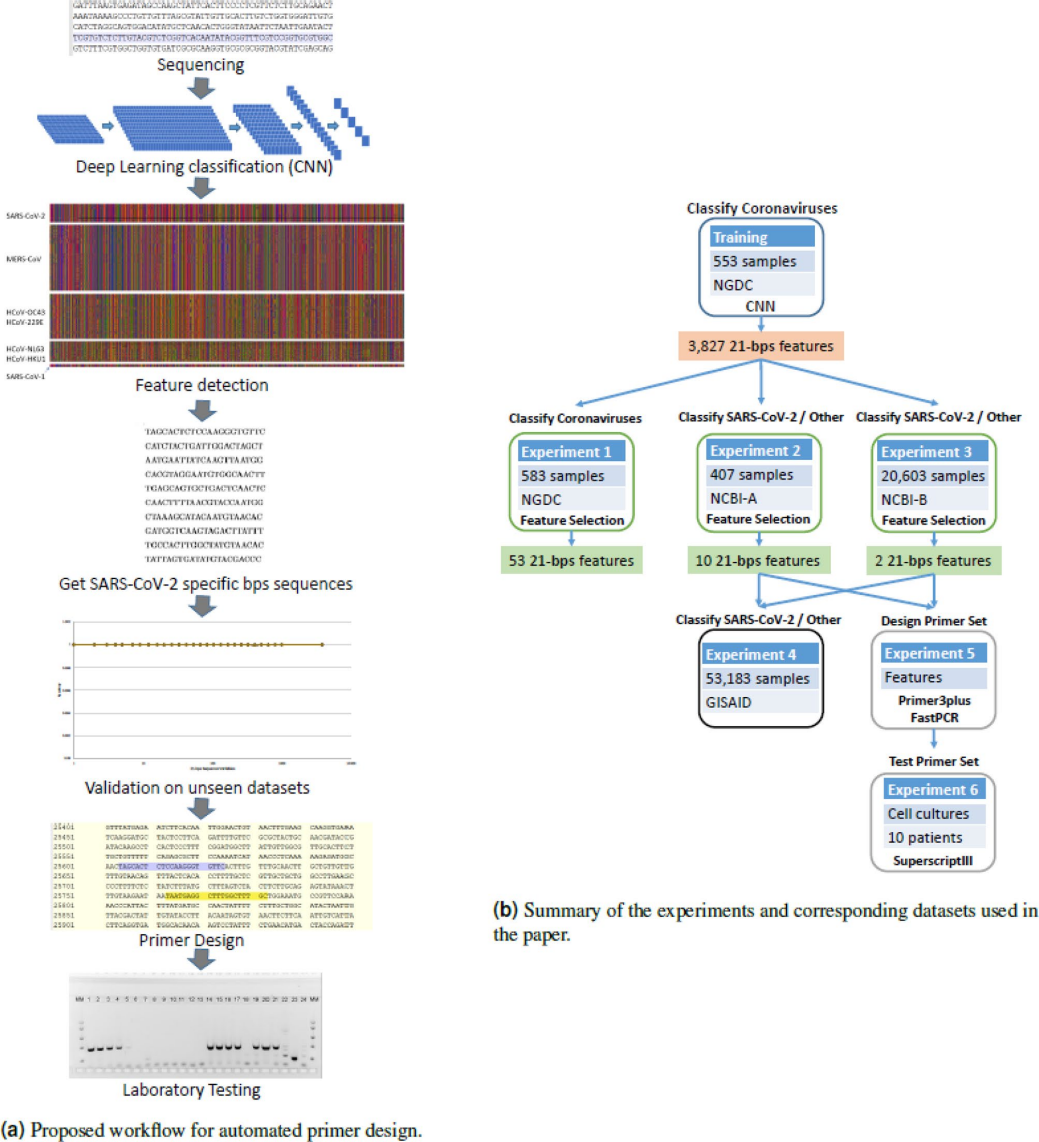
On the left, (**a**) shows the proposed workflow for the automated design of primers for viruses. On the right, (**b**) summarizes the different experiments reported in the paper, along with the datasets used in each trial.

Using a CNN to separate Coronaviruses belonging to different strains^35^, including SARS-CoV-2, we apply techniques inspired by XAI in computer vision to discover representative cDNA sequences that the network uses to classify SARS-CoV-2. We then validate the discovered sequences on datasets not used during the training of the CNN, and show how to exploit them to create a novel, highly informative set of sequence features (e.g. viral sequences). Such sequences can be later inspected and analyzed by human experts. Experimental results show that the new set of sequence features leads traditional, simple classifiers, to correctly assess SARS-CoV-2 with remarkable accuracy (> 99%). A few of the discovered sequences also possess the correct characteristics for potentially becoming primers, as just checking for their presence in samples is enough to specifically identify SARS-CoV-2. Laboratory testing on the most promising sequences identified, showed that the primers found by our approach can be a viable alternative to the commonly adopted primers at the time of writing. These results could pave the way to an automatic procedure for the design of primers, see Fig. 1 for the proposed workflow.

## Results

### CNN classification and feature construction

**Figure 2:**
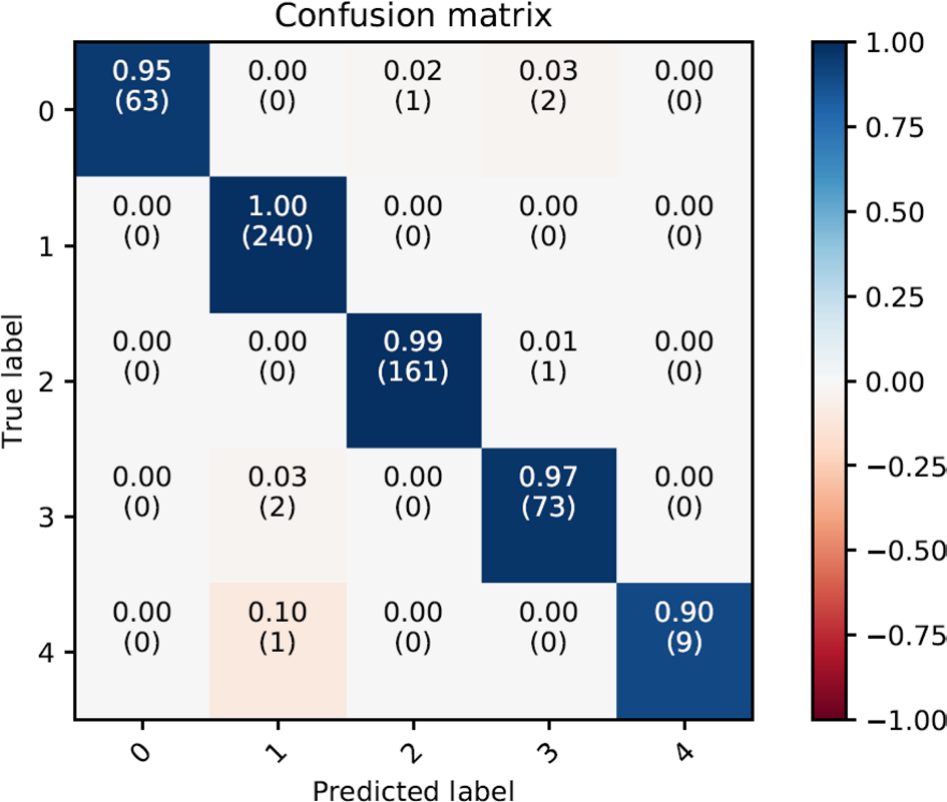
Confusion Matrix of the 10-fold stratified cross-validation for the CNN classifier in the original 553 SARS-CoV-2 sequences.

The trained CNN described in the “Methods” section obtained a mean accuracy of 98.73% in a 10-fold stratified cross-validation. Observing the confusion matrix for the 5 considered classes, reported in Fig. 2 it is remarkable to notice that even samples from underrepresented classes were mostly correctly positioned. Such an encouraging result can indicate that the network was routinely able to uncover meaningful sequences to separate the different classes of viruses.

**Figure 3:**
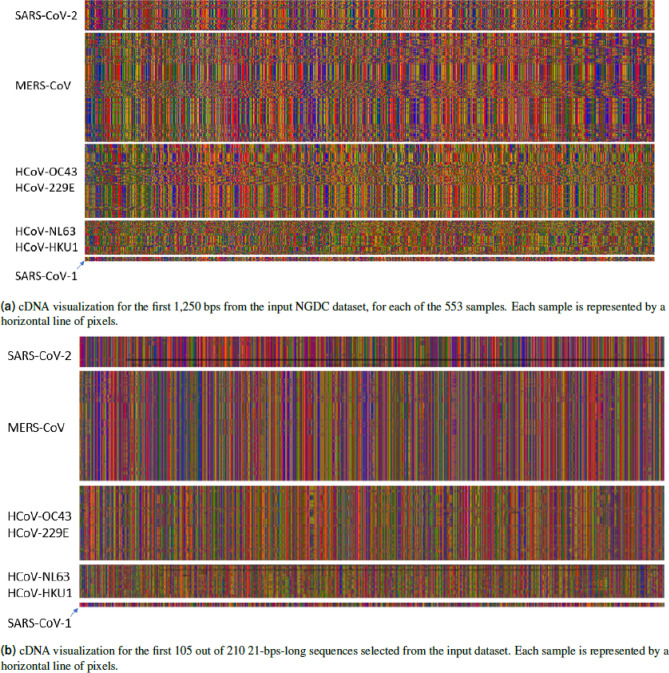
Input 3a, and output 3b of the methodology in colored pixels represent bases: G = green, C = blue, A = red, T = orange, missing = black. The data is separated by class SARS-CoV1: SARS-CoV, SARS-CoV P2, SARS-CoV HKU-39849 and SARS-CoV GDH-BJH01. For visualization purposes we do not show HCov-EMC and HCoV-4408, given the number of samples. From visual inspection, it is possible to notice the similarity of patterns between samples belonging to the same class.

Once the network is trained, in a first analysis, we plot the inputs and outputs of the convolutional layer, to visually inspect for patterns. As an example, in Fig. 3a we report the visualization of the first 1250 bps of each of the 553 samples from the NGDC^11^ repository.

Since each filter in the network slides a 21-bps window over the input, and for each step produces a single value, the output of a filter is a sequence of values in (0, 1). The output of the max pooling for each of the 12 filters is then further inspected for patterns. It is noticeable how samples belonging to different classes can be already visually distinguished. At this step, we identify filter 0 as the most promising, as it seems to focus on a few relevant points in the genome, that could correspond to meaningful cDNA sequences.

Given this data, it is now possible to identify the 21-bps sequences that obtained the highest output values in the max pooling layer of filter 0, in a section of 148 positions. This process results in 210 (31,029 divided by 148) *max pooling features*, each one identifying the 21-bps sequence that obtained the highest value from the convolutional filter, in a specific 148-position interval of the original genome: the first max pooling feature will cover positions 1–148, the second will cover position 149–296, and so on. We show the complete set of max pooling features for the complete data 4410 (210*21) arranged one after the other, in Fig. 3b. The CNN architecture is described in the methods section, the visualization of the filter, and max pooling are available in the Supplementary Materials, Section 1.

Analyzing the different sequence values appearing in the max pooling feature space, we get a total of 3827 unique 21-bps cDNA sequences, that can potentially be informative for identifying different virus strains. For example, sequence **AGG TAA CAA ACC AAC CAA CTT** is only found inside the class of SARS-CoV-2, in 59 out of the 66 available samples. Sequence **CAC GAG TAA CTC GTC TAT CTT** is present again only in SARS-CoV-2, in 63 out of the 66 samples.

The combination of the convolutional and max pooling layer allows the CNN to identify sequences even if they are slightly displaced in the genome (by up to 148 positions in the genome). Thus, we create a table of feature appearance of each of the sequences selected from the previous step. This results in a set of features able to differentiate SARS-CoV-2 from other viruses.

The experiments presented in the following subsections to validate our method have different objectives and make use of different datasets. A summary of all the experiments and datasets used is shown in Fig. 1.

### Identifying SARS-CoV-2

Recapitulating the results of experiments 1–4 (see Fig. 1), we discovered 12 meaningful 21-bps sequences that best characterize SARS-CoV-2. For all the analyzed data, these sequences appear only in SARS-CoV-2 samples and not in any other viruses, as summarized in Table 1. Remarkably, our results outperform earlier publications using machine learning for identifying SARS-CoV-2 (see for example^26^), with the added benefit of producing human-readable results instead of a plain black box classifier.

**Table 1:** Percentage of appearance for each of the 12 discovered 21-bps sequences across the different datasets, and comparison to similar viruses in nature and other hosts.

Source	GISAID	NCBI	NCBI	NGDC	NGDC	GISAID	GISAID	GISAID	GISAID
Virus	SARS-CoV-2	Other Taxa	SARS-CoV-2	Other Taxa	SARS-CoV-2	Betacoronavirus	Betacoronavirus	Betacoronavirus	Betacoronavirus
Host	Homo Sapiens	Homo Sapiens	Homo Sapiens	Homo Sapiens	Homo Sapiens	Manis javanica	Rhinolophus affinis	Canine	Felis Catus
# Samples	52,645	20,572	32	487	96	17	1	2	6
$^1$CAC GTA GGA ATG TGG CAA CTT	99.84%	0.00%	100.00%	0.00%	97.92%	0.00%	100.00%	100.00%	50.00%
$^1$TAT TAG TGA TAT GTA CGA CCC	99.73%	0.00%	100.00%	0.00%	97.92%	0.00%	0.00%	100.00%	50.00%
$^1$AAT GAA TTA TCA AGT TAA TGG	99.94%	0.00%	100.00%	0.00%	96.88%	76.47%	0.00%	100.00%	66.67%
$^1$CAA CTT TTA ACG TAC CAA TGG	99.55%	0.00%	100.00%	0.00%	97.92%	0.00%	0.00%	100.00%	50.00%
$^1$CTA AAG CAT ACA ATG TAA CAC	99.76%	0.00%	100.00%	0.00%	100.00%	0.00%	0.00%	100.00%	66.67%
$^1$TAG CAC TCT CCA AGG GTG TTC	99.57%	0.00%	100.00%	0.00%	97.92%	0.00%	0.00%	100.00%	66.67%
$^1$TGC CAC TTG GCT ATG TAA CAC	99.90%	0.00%	100.00%	0.00%	97.92%	0.00%	100.00%	100.00%	66.67%
$^1$CAT CTA CTG ATT GGA CTA GCT	99.79%	0.00%	100.00%	0.00%	97.92%	0.00%	100.00%	100.00%	50.00%
$^1$TGA GCA GTG CTG ACT CAA CTC	99.56%	0.00%	100.00%	0.00%	98.96%	0.00%	0.00%	100.00%	66.67%
$^1$GAT GGT CAA GTA GAC TTA TTT	99.69%	0.00%	100.00%	0.00%	96.88%	0.00%	0.00%	100.00%	66.67%
$^2$AAT AGA AGA ATT ATT CTA TTC	99.73%	0.00%	100.00%	0.00%	96.88%	0.00%	100.00%	100.00%	66.67%
$^2$CGA TAA CAA CTT CTG TGG CCC	99.06%	0.00%	100.00%	0.00%	97.92%	0.00%	100.00%	50.00%	50.00%

$^1$21-bps sequence resulting from experiment 2.

$^2$21-bps sequence resulting from experiment 3.

### Laboratory validation of the candidate primer set

We calculated the frequency of appearance of different primer sets’ sequences used in SARS-CoV-2 RT-PCR tests developed by World Health Organization (WHO) referral laboratories, and compared it to our primer design on the GISAID dataset (Table 2). All of the sequences have a frequency of appearance of $>99\%$, with the exception of CHINA-CDC-N-F, with a 68.52%. This is consistent with the percentage of genomes with mutation in the primer region, as stated by latest GISAID update summary of August 11th, 2020^31^. For the in-silico analysis of specificity, we compared all the primers sets’ sequences with the NCBI-B and NGDC dataset, and the results show that HKU-N-F, HKU-N-R, Charite-E-F, Charite-E-R and US-CDC-N2-F are not specific to SARS-CoV-2, as they bind to SARS-CoV, too. The rest of the sequences, including our design, only appear in SARS-CoV-2. In summary, of the 8 different primer sets, 3 of them appear to not be specific to SARS-CoV-2; and by frequency of appearance, our design is the 3rd best option among the remaining 5, considering the lowest frequency between the -F and -R primer. This is a remarkable result, considering that the proposed primer set has been extracted by an almost completely automated procedure.

**Table 2:** Comparison of primer sets developed by WHO referral labs, against the primer set obtained by the proposed approach, listed as *UtrechtU-ORF3a*.

Primer	Sequence	Frequency (%)	Specific (Yes/No)
Charite-E-F	5′-ACA GGT ACG TTA ATA GTT AAT AGC GT-3′	99.90	No
Charite-E-R	5′-ATA TTG CAG CAG TAC GCA CAC A-3′	99.90	No
CHINA-CDC-ORF1ab-F	5′-CCC TGT GGG TTT TAC ACT TAA-3′	99.90	Yes
CHINA-CDC-ORF1ab-R	5′-ACG ATT GTG CAT CAG CTG A-3′	99.59	Yes
HKU-N-F	5′-TAA TCA GAC AAG GAA CTG ATT A-3′	99.56	No
HKU-N-R	5′-CGA AGG TGT GAC TTC CAT G-3′	99.58	No
US-CDC-N1-F	5′-GAC CCC AAA ATC AGC GAA AT-3′	99.71	Yes
US-CDC-N1-R	5′-TCT GGT TAC TGC CAG TTG AAT CTG-3′	99.57	Yes
US-CDC-N2-F	5′-TTA CAA ACA TTG GCC GCA AA-3′	99.43	No
US-CDC-N2-R	5′-GCG CGA CAT TCC GAA GAA-3′	99.74	Yes
UtrechtU-ORF3a-F	5′-TAG CAC TCT CCA AGG GTG TTC-3′	99.57	Yes
UtrechtU-ORF3a-R	5′-GCA AAG CCA AAG CCT CAT TA-3′	99.48	Yes
US-CDC-N3-F	5′-GGG AGC CTT GAA TAC ACC AAA A-3′	99.09	Yes
US-CDC-N3-R	5′-TGT AGC ACG ATT GCA GCA TTG-3′	99.72	Yes
CHINA-CDC-N-F	5′-GGG GAA CTT CTC CTG CTA GAA T-3′	68.52	Yes
CHINA-CDC-N-R	5′-CAG ACA TTT TGC TCT CAA GCT G-3′	99.20	Yes

The Frequency column indicates frequency of appearance of the sequence among samples of the GISAID dataset. The Specific (Yes/No) column shows whether the sequence appears to be unique to SARS-CoV-2 samples, or can also be found in other viruses, according to the evaluation on the NCBI-B and NGDC datasets.

**Figure 4:**
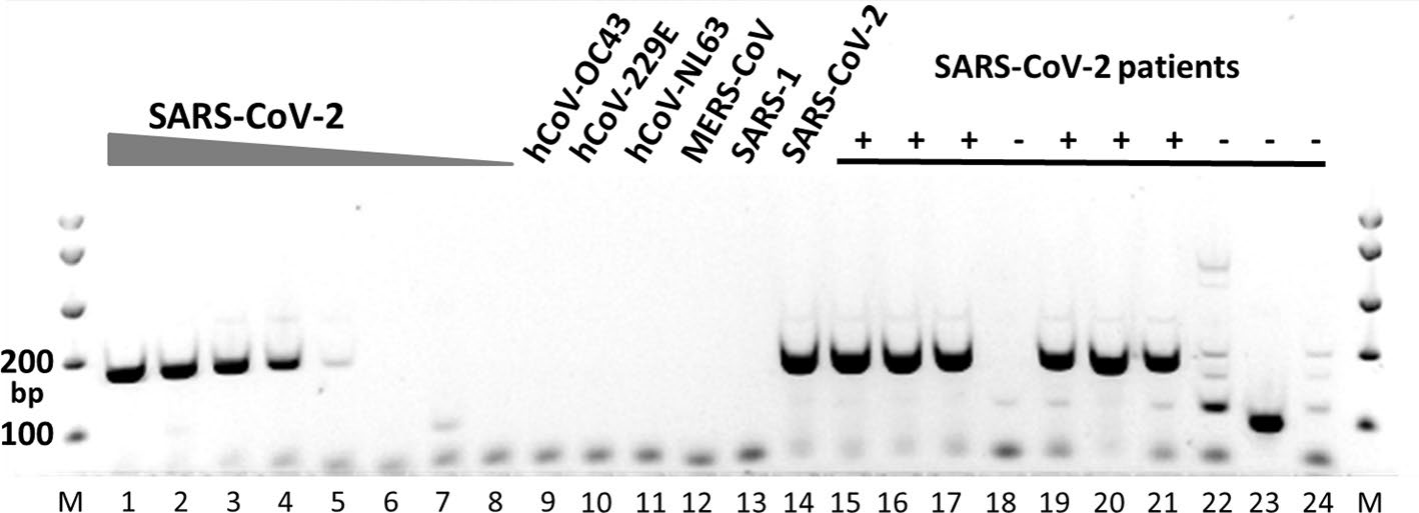
Laboratory validation of the candidate primer set by conventional PCR. MM, molecular weight marker; lanes 1–8, 10-fold dilutions of SARS-CoV-2 RNA (corresponding to Ct values 26–39 in the diagnostic reference assay); lanes 9–14, RNA from different human coronaviruses (hCoV-OC43, hCoV-229E, hCoV-NL63, MERS-CoV, SARS-1, SARS-CoV-2, respectively); lanes 15, 16, 17, 19, 20, 21, patient samples previously found positive for SARS-CoV-2; lanes 18, 22, 23, 24, patient samples previously found negative for SARS-CoV-2.

To validate the data obtained in-silico by laboratory methods, a conventional PCR was performed on cDNA obtained from RNA from SARS-CoV-2 and other human coronaviruses. In addition, RNAs from nasopharyngeal swabs from six patients previously diagnosed with SARS-CoV-2 infection and four patients negative for SARS-CoV-2 by routine diagnostic method^5^ were analyzed with the same conventional PCR (Fig. 4). Different dilutions of SARS-CoV-2 RNA were detected with similar sensitivity compared to the diagnostic reference assay. (Fig. 4, lanes 1–8). Our candidate primer set exclusively detected SARS-CoV-2 and did not amplify RNA from other human coronaviruses (Fig. 4, lanes 9–14). The candidate primer set was able to detect SARS-CoV-2 RNA from patient samples previously found positive for SARS-CoV-2, but not in patients previously found negative (Fig. 4, lanes 15–24). Although further validation will be required to develop this candidate primer set into a diagnostic assay, our results clearly demonstrate the power of our method to select potential sequences for further validation.

## Discussion

Being able to reliably identify SARS-CoV-2 and distinguish it from other similar pathogens is important to contain its spread. The time of processing samples and the availability of reliable diagnostic tests is a challenge during an outbreak. Developing innovative diagnostic tools that target the genome to improve the identification of pathogens, can help reduce health costs and time to identify the infection, instead of using unsuitable treatments or testing. Moreover, it is necessary to perform an accurate classification to identify the different species of Coronavirus, the genetic variants that could appear in the future, and the co-infections with other pathogens.

Given the high transmissibility of the SARS-CoV-2, the proper diagnosis of the disease is urgent, to stop the virus from spreading further. Considering the false negatives given by the standard RT-qPCR detection, better implementations such as using deep learning are necessary in order to properly detect the virus. While the accuracy of current RT-qPCR testing is around 70%, and CT scans with deep learning go up at 83%, we believe that the use of the sequences detected by a CNN-based methodology has the potential to improve the accuracy of the diagnosis.

Our results, show that by targeting one out of the 12 selected 21-bps specific sequences, we are able to distinguish SARS-CoV-2, from any other virus (> 99%). Further testing is necessary to confirm these promising results so it is essential to create multidisciplinary groups that work to stop the outbreak. Finally, as an interesting remark, by comparing the discovered sequences against other hosts, we noticed that from the 12 sequences exclusive to SARS-CoV-2, one of them appears in 13 of 17 samples from *Manis Javanina*. In contrast, 5 of the sequences of SARS-CoV-2 appear in the only sample available from *Rhinolophus Affinis* and 11 out of 12 in 2 *Canine* samples (Table 1). This is consistent with the findings of Zhang et al.^36,37^, and could point to the zootonic origin of the virus. Nevertheless, more data is necessary.

As a result of the high density populations, and ever growing interaction between people, it is possible that other pandemics may occur. We believe that our methodology has a substantial added value over traditional methods, because it is a fast method and only limited set of viral sequencing data is needed. Moreover, this procedure led to a primer set with a very high specificity for SARS-CoV-2 with at least the same accuracy as the best primers sets in the world developed by WHO referral laboratories. Thus, thinking forward, our methodology can be applied in future viral pandemics to speed up the development of accurate detection methods for diagnosis and thereby contribute to limit the spread of a virus.

## Methods

**Figure 5:**
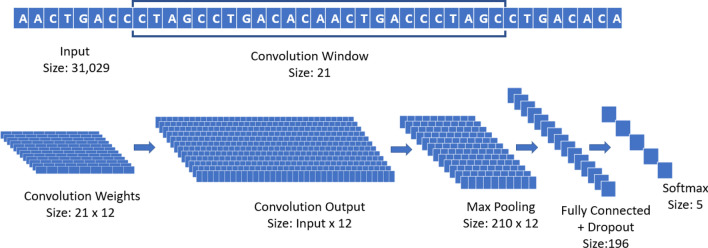
Graphical representation of the architecture of the CNN used in the experiments.

The CNN used during all the experiments is composed of one convolutional layer with 12 different filters or weights (each with window size 21, and an even padding of 10 steps on each side) with maxpooling (pool size 148 and stride 1), a fully connected layer (196 rectified linear units with dropout probability 0.5), and a final softmax layer with 5 units, to differentiate the different classes of Coronavirus strains. The optimizer used is Adaptive Momentum (ADAM)^38^, with learning rate $10^{-5}$ and a batch size of 50 samples, run for 1000 epochs^35^. A graphical summary of the CNN used in the experiments is reported in Fig. 5.

The convolutional layer of the network, in simple terms, is analyzing subsequences of 21 base pairs that can appear in different points of the virus genome. We selected 21 as designed primers for RT-PCR tests have a length of 18–22 bps normally. The pool size of the maxpooling represents the interval in which a specific 21-bps sequence can be recognized (in this case, 148 positions). Through the training process, the convolutional layer is de-facto learning new features to characterize the problem, directly from the data. In this specific case, the new features are 21-bps sequences that can more easily separate different virus strains. By analyzing the result of each filter in a convolutional layer, and how its output interacts with the corresponding max pooling, it is possible to detect human-readable sequences of base pairs that might provide domain experts with relevant information. It is important to notice that these sequences are not bound to specific locations of the genome; thanks to its structure, the CNN is able to detect them and recognize their importance even if their position is displaced in different samples.

We downloaded 583 sequences (*.fasta files) from the NGDC on March 15th, 2020 (Table 3). We divided the samples into 5 classes, taking into account both the number of available samples, and the seasonality of the related diseases. SARS-CoV-2 on its own as class 0, as the main objective is to separate this virus from all the others. The 240 samples from MERS-CoV/HCoV-EMC were assigned to class 1, as the related disease is mostly geographically limited to areas of Saudi Arabia^39^. HCoV-OC43, HCoV-229E and HCoV-NL63 have been reported to have winter seasonality, while HCoV-HKU1 has spring-summer seasonality. Nevertheless, there are instances where HCoV-NL63 had a spring-summer seasonality closer to HCoV-HKU1^40–42^. Thus, also considering the number of samples, we grouped together HCoV-OC43, and HCoV-229E as class 2, while HCoV-HKU1 and HCoV-NL63 were grouped as class 3. We included HCoV-4408 in class 2 as well, as there were only 2 samples available: HCoV-4408 is subgroup A of the betacoronavirus genus, as HCoV-OC43^43^. Finally, as we deemed important to distinguish SARS-CoV-2 from SARS-CoV, SARS-CoV was assigned to its own class (class 4) even if only 10 samples were available.

We left out 30 SARS-CoV-2 sequences and then performed a 10-fold stratified cross-validation, with the remaining data divided into 80% training (8 folds), 10% validation (1 fold), 10% testing (1 fold). The stratified cross-validation preserves the ratio of classes in the original dataset for each fold, thus making the final accuracy more reliable.

### Identifying SARS-CoV-2

#### Experiment 1: Validation on the NGDC dataset

We downloaded the dataset from the NGDC repository^11^ on March 15th, 2020. We removed repeated sequences and applied the procedure to translate the data into the sequence feature space. This leaves us with a frequency table of 3827 features (21-bps sequences) with 583 samples (Table 3 (left)). Next, we ran a state-of-the-art feature selection algorithm^45,46^, to reduce the sequences needed to identify different virus strain to the bare minimum. Remarkably, we are then able to correctly differentiate all the coronavirus (MERS-CoV, SARS-CoV-2, SARS-CoV-1, etc) samples using only 53 of the original 3827 sequences, obtaining a 100% accuracy in a 10-fold cross-validation with a simpler and more traditional classifier, such as Logistic Regression. The list of the 53 features is available in the Supplementary Materials, Section 2.

**Table 3:** Organism, assigned label, and number of samples in the unique sequences for the NGDC repository (left) and query: *gene = “ORF1ab” AND host = “homo sapiens” AND “complete genome”* in the NCBI repository (right).

Organism	Label	Number of samples	Organism	Label	Number of samples
SARS-CoV-2	0	96	SARS-CoV-2	0	68
MERS-CoV	1	236	MERS-CoV	1	180
HCoV-EMC	1	4	HCoV-HKU1	1	13
HCoV-OC43	2	138	HCoV-OC43	1	105
HCoV-229E	2	22	HCoV-NL63	1	29
HCoV-4408	2	2	HCoV-4408	1	2
HCoV-NL63	3	58	HCoV-229E	1	3
HCoV-HKU1	3	17	HCoV-EMC	1	3
SARS-CoV	4	7	HAstV-VA1	1	1
SARS-CoV P2	4	1	HAstV-BF34	1	1
SARS-CoV HKU-39849	4	1	HMO-A	1	1
SARS-CoV GDH-BJH01	4	1	HAstV-SG	1	1
Total samples	–	583	Total samples	–	407

We use the NCBI organism naming convention^44^.

#### Experiment 2: Validation on the NCBI dataset

We downloaded data from the NCBI^30^ repository on March 15th, 2020, with the following query: *gene = “ORF1ab” AND host = “homo sapiens” AND “complete genome”*. The query resulted in 407 non-repeated sequences (Table 3 (right)). We call this dataset NCBI-A, where 68 sequences belong to SARS-CoV-2. Then, we applied the procedure to translate the data into the set of sequence features, and we run the same state-of-the-art feature selection algorithm^45^. The result is a list of 10 different sequences (Table 1), for which just checking for their presence is enough to differentiate between SARS-CoV-2 and other viruses in the dataset, with a 100% accuracy. Each of the sequences, in fact, only appears in SARS-CoV-2 samples.

#### Experiment 3: Further validation on the NCBI dataset

We downloaded data from NCBI^30^ on March 17th, 2020, with the following query: *“virus” AND host = “homo sapiens” AND “complete genome”*, restricting the size from 1000 to 35,000 bps (NCBI-B). The query returns 20,603 samples, of which only 32 belong to SARS-CoV-2, and 20,571 are from other taxa, including Hepatitis B, Dengue, Human immunodeficiency, Human orthopneumovirus, Enterovirus A, Hepacivirus C, Chikungunya virus, Zaire ebolavirus, Human respirovirus 3, Orthohepevirus A, Norovirus GII, Hepatitis delta virus, Mumps rubulavirus, Enterovirus D, Zika virus, Measles morbillivirus, Enterovirus C, Human T-cell leukemia virus type I, Yellow fever virus, Adeno-associated virus, rhinovirus (A, B and C), for a total of more than 584 other viruses (not considering strains and isolates). Then, we applied the procedure to translate the data into the sequence feature space and run the feature reduction algorithm^45^. This results in 2 extra sequences of 21 bps: just by checking for their presence, we are able to separate SARS-CoV-2 from the rest of the samples with a 100% accuracy (Table 1).

#### Experiment 4: Validation on the GISAID dataset

From the GISAID repository^31^, we downloaded 53,183 sequences available on August 10th, for SARS-CoV-2, from different countries, from there 52,645 have as $<1\%$ Ns, high coverage and *host = “homo sapiens”*. Then, we calculated the frequency table of the 21-bps sequences obtained from experiments 2 and 3, to verify which sequences remain and could be used for detection. The appearance frequency of the target sequences among the samples in the GISAID dataset is reported in Table 1, second column. In addition, we downloaded 26 sequences from GISAID repository of other hosts (*manis javanica*, *rhinolophus affinis*, *canine* and *felis catus*) to make a comparison in the sequences from experiment 2 and 3.

### Laboratory validation of the candidate primer set

#### Experiment 5: Design of the candidate primer set

After the analysis carried out on the deep learning model, we ran an analysis with Primer3plus^47^, to see which of the sequences could be used as a forward primer, using sample NCBI NC045512.2 as the reference SARS-CoV-2 sequence. We uncover the sequence **TAG CAC TCT CCA AGG GTG TTC** that shows a frequency of appearance of 99.57% in viral genomes available from different countries in GISAID^31^ and 100.0% in the NCBI^30^ datasets. Using the reference SARS-CoV-2 sequence, we identify that this discovered sequence is located between nucleotides 25,604 and 25,624 in the ORF3a gene. In SARS-CoV, this gene encodes a protein of 274 aa, that is related with necrotic cell death^48,49^, chemokine production like interleukin 8 (IL-8) and RANTES/CCL5, NF$\kappa B$ activation resulting in an inflammatory response^50^ and may play an important role in the virus life cycle^51^. We design a specific primer set for detection of SARS-CoV-2 using Primer3plus^47^. We use **TAG CAC TCT CCA AGG GTG TTC** as forward primer and **GCA AAG CCA AAG CCT CAT TA** as reverse primer, obtaining an amplicon size of 179 bps. Then, we run an *in-silico PCR* test using FastPCR 6.7^52^ with default parameters in NC045512.2 used as a reference SARS-CoV-2 sequence, this yields $Tm=56.2\,^{\circ }$C for the forward primer, $Tm=53.1\,^{\circ }$C for the reverse primer and $Ta=58\,^{\circ }$C.

In addition, we calculated the frequency of appearance of different primers sets’ sequences used in SARS-CoV-2 RT-qPCR tests developed by WHO referral laboratories and compared it to our primer design sequences in 52,645 sequences from the GISAID repository and the 583 samples of different coronaviruses from the NGDC dataset from experiment 1. The used primers set are developed by University of Hong Kong (HKU-N); Charite, Berlin, Germany (Charite-E); US-CDC, United States (US-CDC-N1,US-CDC-N2,US-CDC-N3) and China CDC, China (China-CDC-ORF1ab, China-CDC-N) (Table 2). We selected this primers as they are the ones more commonly used as stated in the GISAID status update of August 11, 2020. We do not consider degenerate primer sets.

#### Experiment 6: Validation of the candidate primer set in biological samples.

Viral RNA was isolated from cell-cultured SARS-CoV-2, SARS-1, MERS-CoV, hCoV-NL63, hCoV-OC43, hCoV-229E, and from nasopharyngeal swabs from $n=10$ patients by MagNA Pure LC (Roche Diagnostics, The Netherlands) using the total nucleic acid isolation kit. The RNA was converted into cDNA using SuperscriptIII (Thermo-Fisher Scientific, USA) and random hexamers. Subsequently, conventional PCR was performed on the cDNA using HotStar Taq DNA polymerase (Qiagen, The Netherlands) with 400 nM forward primer (5′-**AG CAC TCT CCA AGG GTG TTC**-3′) and 400 nM reverse primer (5′-**GCA AAG CCA AAG CCT CAT TA**-3′) and the following cycling conditions: 15 min at 95$^\circ$C, followed by 40 cycles of 1 min at 95$\,^{\circ }$C, 1 min at 5 $^{\circ }$C and 1 min at 72$\,^{\circ }$C. The PCR products were visualized by electrophoresis. The same RNA was used in a diagnostics reference assay by Corman et al.^5^ and the Cycle threshold values form this reference assay were used for estimating sensitivity.

## Supplementary Information

Supplementary material 1

## Data Availability

All the necessary scripts to reproduce the experiments are stored on the public GitHub repository: https://github.com/steppenwolf0/primers-sars-cov-2. Due to storage limits, while the data to reproduce Experiment 1 is included in the repository, for Experiments 2–5 only the IDs of the samples used are listed in file sample-ids.xlsx. Given their IDs, samples for Experiments 2–5 can be downloaded from the corresponding open repositories: : GISAID (https://www.gisaid.org/), NCBI (https://www.ncbi.nlm.nih.gov/), NGDC (https://bigd.big.ac.cn/ncov/?lang=en).
