# Supplementary material for: Classification and specific primer design for accurate detection of SARS-CoV-2 using deep learning
Source: Sci Rep. 2021 Jan 13;11:947. doi: 10.1038/s41598-020-80363-5 (PMC7806918; doi:10.1038/s41598-020-80363-5)
Supplement: Supplementary file 1 — Supplementary material 1 [file 41598_2020_80363_MOESM1_ESM.pdf]

Classification and Specific Primer Design for Accurate Detection of SARS-CoV-2 Using Deep Learning: Supplementary Information

Alejandro Lopez-Rincon[1,*], Alberto Tonda[2], Lucero Mendoza-Maldonado[3], Daphne G.J.C. Mulders[4], Richard Molenkamp[4], Carmina A. Perez-Romero[5], Eric Claassen[6], Johan Garssen[1,7], and Aletta D. Kraneveld[1]

[1] *Division of Pharmacology, Utrecht Institute for Pharmaceutical Sciences, Faculty of Science, Utrecht University, Universiteitsweg 99, 3584 CG Utrecht, the Netherlands*
[2] *UMR 518 MIA-Paris, INRAE, c/o 113 rue Nationale, 75103, Paris, France*
[3] *Hospital Civil de Guadalajara "Dr. Juan I. Menchaca". Salvador Quevedo y Zubieta 750, Independencia Oriente, C.P. 44340 Guadalajara, Jalisco, México*
[4] *Department of Viroscience, Erasmus Medical Center, Rotterdam, the Netherlands*
[5] *Departamento de Investigación, Universidad Central de Queretaro (UNICEQ), Av. 5 de Febrero 1602, San Pablo, 76130 Santiago de Querétaro, Qro., Mexico*
[6] *Athena Institute, Vrije Universiteit, De Boelelaan 1085, 1081 HV Amsterdam, the Netherlands*
[7] *Department Immunology, Danone Nutricia research, Uppsalalaan 12, 3584 CT Utrecht, the Netherlands*
[*] *a.lopezrincon@uu.nl*

1. CNN ARCHITECTURE AND VISUALIZATION

The original samples used in the experiments are virus sequences, e.g. a series of base pairs **[A, C, C, G, T, ...]**. Before the start of the experiments with the CNN, the sequences are converted to numerical values, using the following representation: N (missing) $\rightarrow$ 0.0, C $\rightarrow$ 0.25, T $\rightarrow$ 0.5, G $\rightarrow$ 0.75, A $\rightarrow$ 1.0, so that the sequence in the previous example would become **[1.0, 0.25, 0.25, 0.75, 0.5, ...]**.

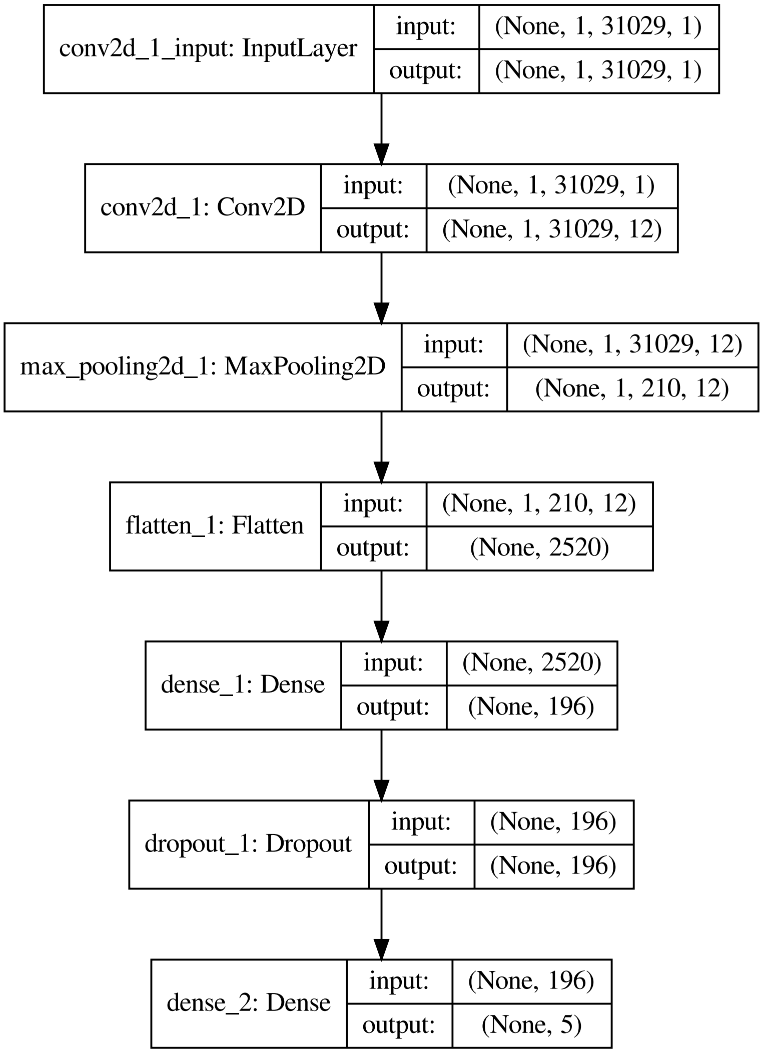

Fig. S1. Shapes of tensors in the CNN used in the experiments.

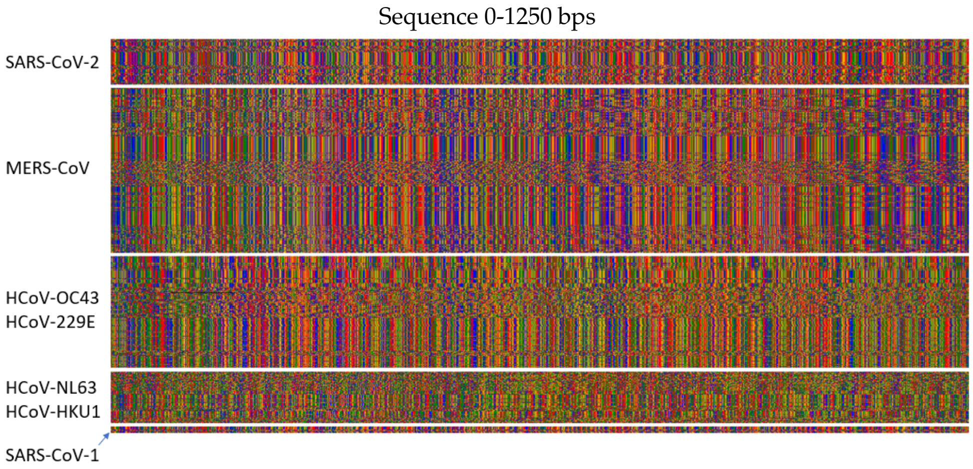

Fig. S2. cDNA visualization for the first 1,250 bps from the input NGDC dataset, for each of the 553 samples. Each sample is represented by a horizontal line of pixels. Colored pixels represent bases: G=green, C=blue, A=red, T=orange, missing=black. The data is separated by class SARS-CoV1: SARS-CoV, SARS-CoV P2, SARS-CoV HKU-39849 and SARS-CoV GDH-BJH01. For visualization purposes we do not show HCov-EMC and HCoV-4408, given the number of examples.

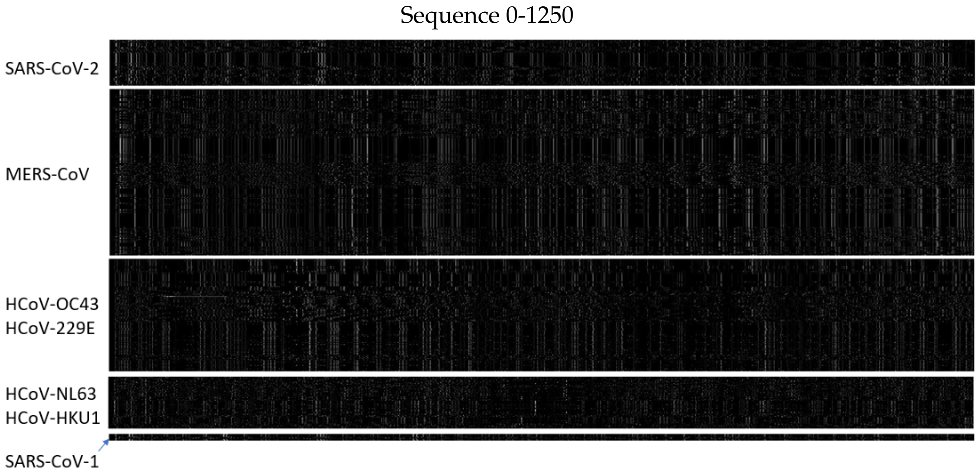

Fig. S3. The output of convolutional filter 0, for the input given in Fig. S2. The output of the filters is a series of continuous values in $(0, 1)$, here represented in grayscale, with higher values closer to white.

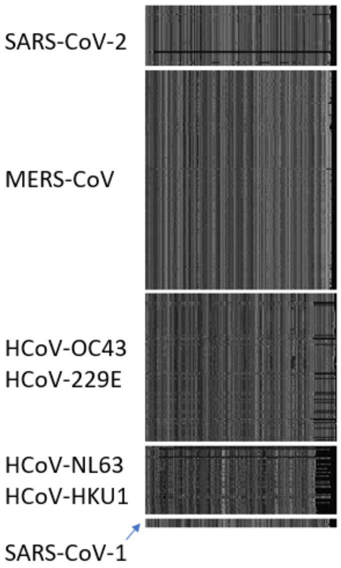

Fig. S4. Visualization of the output of the max pooling for the first filter of the CNN, with the data visualized in Fig. S3 in input. Different patterns for samples from different classes are recognizable from a simple visual inspection. Here samples from the same class appear grouped, one after the other, for clarity.

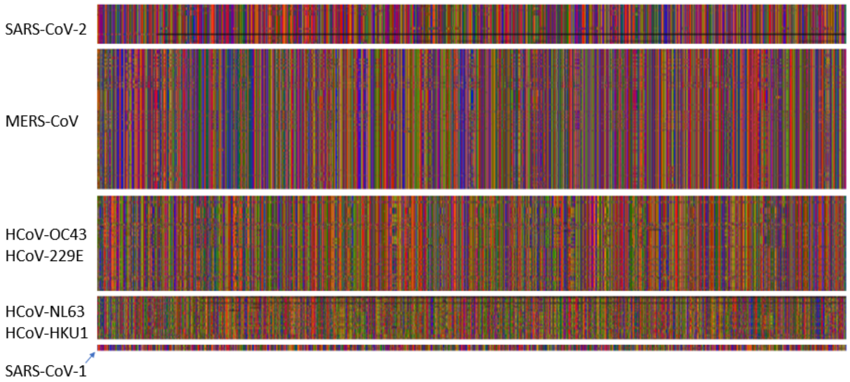

(a) 0 - 2,205 bps

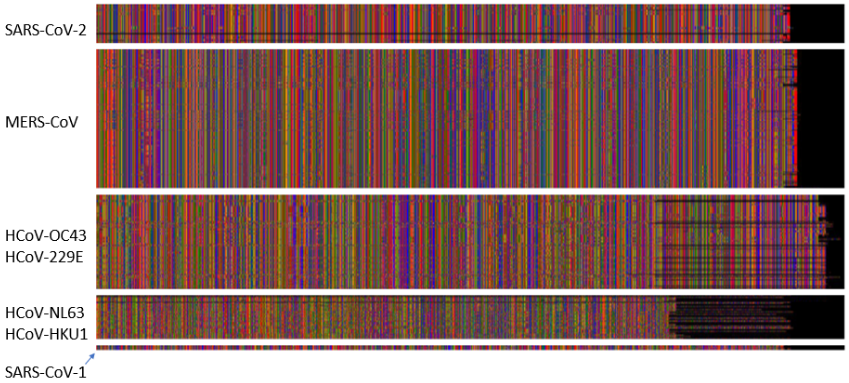

(b) 2,205 - 4,410 bps

Fig. S5. cDNA visualization for the selected 210 21-bps-long sequences selected from the input dataset. Each sample is represented by a horizontal line of pixels. Colored pixels represent bases: G=green, C=blue, A=red, T=orange, missing=black. We divide the whole information, for visualization purposes; from visual inspection we can see the similarity of the patterns between the classes.

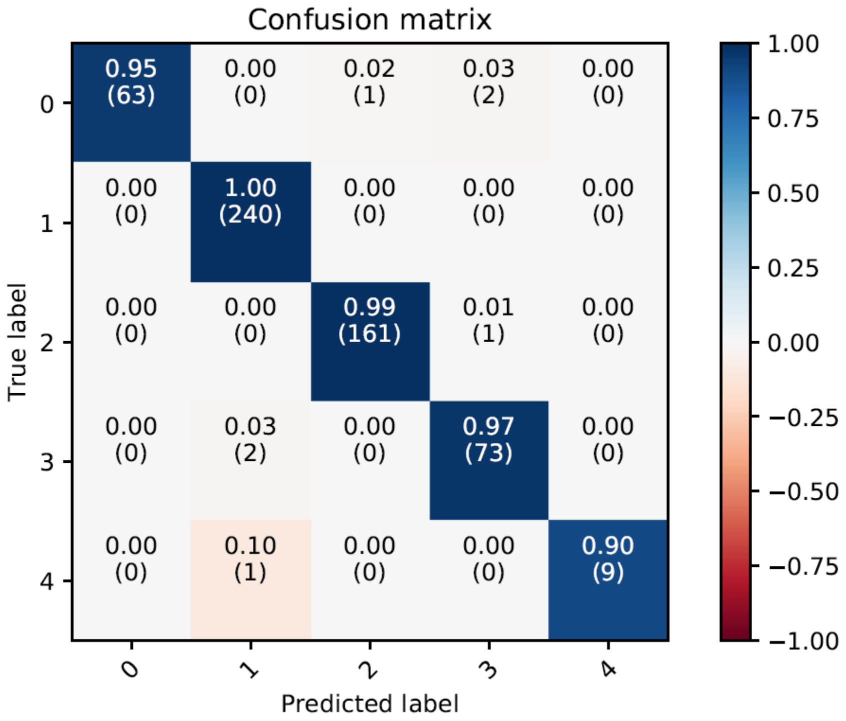

Fig. S6. Confusion Matrix of the 10-fold cross-validation in the original 553 SARS-CoV-2 sequences.

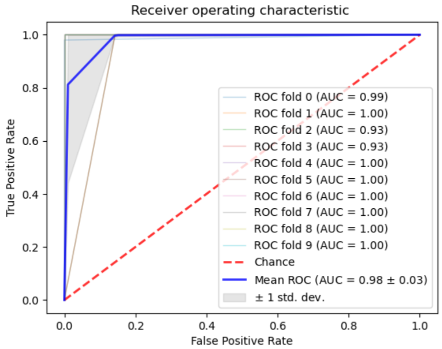

(a) Class 0.

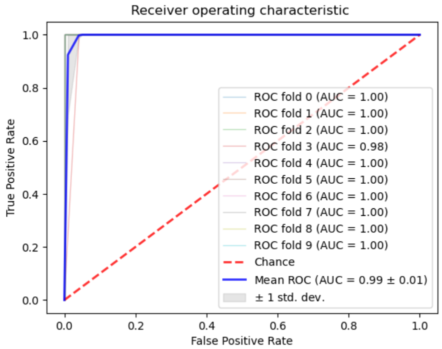

(b) Class 1.

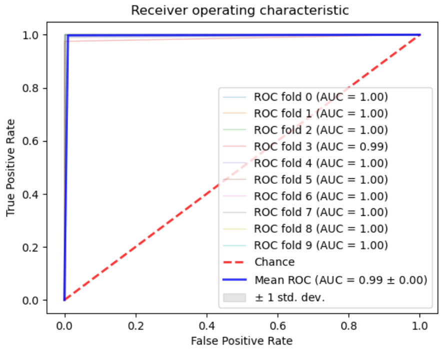

(c) Class 2.

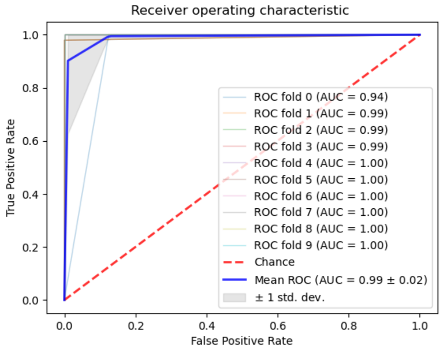

(d) Class 3.

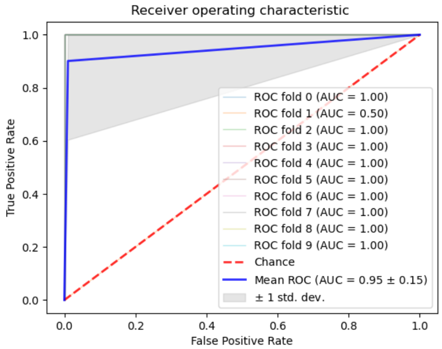

(e) Class 4.

Fig. S7. Resulting Binary ROC curves, for each of the classes in 10-fold cross-validation from the CNN model.

2. SEQUENCES USED TO GENERATE THE ORIGINAL MODEL

Table S1. IDs of the 553 sequences to create the CNN model.

NC_045512	MF542265	KY688124	KF600647	KF923912	CNA0007332	MH940245	MG546330	KT121581	KF923896	KF514432	MT152824
MN120514	KX154693	MK796425	KF600630	KF923913	CNA0007334	MH310910	MF000458	KT121578	KJ958219	KF530090	MT050493
MN120513	KX154694	KY688123	KF600644	KF923914	CNA0007335	MG912598	MG757603	KT121579	KJ958218	KF530074	MT012098
MN365233	KX154692	KT806046	KF600645	KF923915	GWHABKK00000001	MG912596	MF000459	KJ813439	KU131570	KF530097	
MN365232	KX154691	KY688119	KF186567	KF923916	GWHABKL00000001	MG366483	MG757601	KT121580	KX344031	KF530071	
MN306041	MF374983	KT806047	KJ361501	KF923917	GWHABKM00000001	MG912597	KU521535	KM027262	KF923925	KF530079	
MN310476	KX154684	KT026454	KJ361502	KF923891	GWHABKN00000001	MK062184	MG757600	KT861628	KF923888	KF530094	
MN306040	KX154685	KT026456	KJ361503	KF923892	GWHABKO00000001	MK062183	MG757602	KM027260	KF923893	KF530067	
MN306036	KX154686	KT806045	KF600627	KF923894	NMDC60013002-05	MG912595	KX179500	MK039552	KF923895	KF530082	
MN306043	KX154687	KT806049	KF745068	KF923907	NMDC60013002-06	MK062182	MF000460	KY581693	KF923924	KF530095	
MN310478	KX154688	KT806054	KJ156866	KF923921	NMDC60013002-07	MK062181	KU851863	KP209309	MG428701	KF530089	
MN306042	MG757604	KT806055	KF186564	KF923890	NMDC60013002-09	MK062180	MG757598	KM027261	MG428703	KF530091	
MN306046	KX154689	KT026453	KJ361500	KY014282	NMDC60013002-10	MK062179	MH029552	KY581692	KP198610	KF530107	
MN306053	KX154690	KT806044	KF186565	KF923900	MT007544	MH310912	MG757599	KM027259	MG428706	KF530096	
MK129253	KY621348	KT806048	KF186566	KF923899	MN938384	MH310909	MF000457	KP209313	MG428705	KF530076	
MK462256	KY554971	KY688118	KJ156952	KF923889	MT020781	MH432120	MG757594	KM027258	MG428704	KF530113	
MK462255	KY554970	KT806051	KX538979	JQ765575	LC521925	MG011360	MG757597	KY581690	MG428699	KF530104	
MK334046	KY554968	KT806052	KM210277	JQ765574	MT027062	MF374985	KU851860	KP209307	MG428702	KF530109	
MK462254	KY684760	KT806053	KM210278	KF923905	MT027064	MG011361	KU851861	KP209308	KF923918	KF530088	
MK462253	KY369911	KX179496	KM015348	JQ765573	MT039890	MF374984	KU851862	KY581688	KF923887	KF530061	
MH822886	KY369908	KX179499	KF600613	HM034837	MT039888	MK167038	KU851864	KY581689	KF923886	KF530065	
MK483839	KY369912	KR011265	KX538978	JQ765572	MT039887	MG011362	MG757596	KY581686	KF430201	KF530066	
MK334043	KY369913	KX179498	KF192507	JQ765571	CNA0007333	MH121121	MG757595	KP209306	KF686341	KF530111	
MK334045	KY369914	KR011263	KF600652	JQ765570	MT044257	MG977452	MG757593	KM027257	KF686346	KF530073	
MK462252	KY674917	KT266906	KF600612	JQ765569	MT044258	MG977451	KU851859	KP209310	KF686343	KF530108	
MK334047	KY674916	KY673148	KC667074	JQ765568	MT049951	MH454272	KX034100	KT861627	KP198611	KF530114	
MK462251	KY674918	KT381875	KC164505	JN129834	LC522973	MH395139	KT374050	KM027255	JX503060	FJ415324	
MK334044	KY674915	KU291448	KX538977	JN129835	LC522974	MG011358	KX034099	KM027256	KF686344	KF530106	
MK462250	KY674914	KY967360	KX538976	FJ882963	LC522975	MG011357	KT374057	KY581694	KF686342	KF530083	
MK462249	KY554974	KY829118	KX538975	JX504050	LC522972	MH306207	KX034098	KT156561	KF686340	KF530077	
MK462248	KY554973	KY967356	KX538974	GU553363	MT066175	MG011352	KT374056	KY581687	KT779555	KF530087	
MK462247	KY674941	KY967357	KX538973	KY014281	MT066176	MG011356	KT374055	KP209312	KT779556	KF530086	
MN026164	KY674943	KY967358	KX538972	KF530112	MT072688	MG011355	KX034096	KJ556336	FJ938067	KF530085	
MN369046	KY674921	KY967359	KX538971	KF530105	MT081059	MG011354	KX034097	KF958702	JQ765563	KF530060	
MN306018	KY554967	KY983585	KX538970	KF530092	MT081066	MG011353	KT225476	KT156560	JQ765567	KF530110	
MK462246	KY554972	KY983587	KF600620	KF530068	MT093571	MG011348	MG366883	KF961222	JQ765564	AY274119	
MK462245	KY684759	KY983583	JX869059	KF530070	MT093631	MG757605	KT374053	KF961221	KF923922	AY597011	
MK462244	KY674942	KY983586	KF923897	KF530081	MT106053	MG011349	KX034094	KJ156874	JQ765565	DQ640652	
MK462243	KY554975	KY983588	KX538969	KF530069	MT106052	MG011351	KU308549	KJ156934	JX524171	MN975262	
MG912605	KY369909	KY967361	KX538968	KF530080	MT106054	MG011350	MG366881	KJ156910	JX503061	GWHABKF00000001	
MG912604	KY674919	MG772808	KX538967	KF530072	MT118835	MG011359	MG366882	KJ156869	JX104161	GWHABKG00000001	
MG912606	KY674920	KU710264	KF923902	KF530099	MT123290	MG011345	KX034095	KY581685	KF923923	GWHABKH00000001	
MG912608	KY554969	KU710265	KF923903	KF530063	MT123291	MG011346	MG366880	KY581684	JQ765566	GWHABKI00000001	
MG912603	KY369910	KP223131	KF923904	KF530078	MT123292	MG011347	KT374052	KJ156944	KF923919	GWHABKJ00000001	
MG912602	KY369905	MK052676	KX538966	KF530064	MT123293	MG011344	KT006149	KJ156949	KF923920	MN988713	
MG912607	KY369906	KT121576	KC776174	KF530098	LC528232	MG011343	KY688122	KJ156881	KF923901	MN985325	
MG912601	KY369907	KT121572	KX538965	KF530075	LC528233	MF314143	KT326819	KF600628	KF923908	MN994467	
MG912600	MG546331	KT121577	KF923898	KF530084	MT126808	MG011342	KY688121	KF600634	KF923909	MN994468	
MG912599	MG520076	KJ829365	KF923906	KF514430	MT135041	MG011341	MK280984	KF600632	KF923910	MN997409	
MH310911	MH013216	KT121574	KX538964	KF514433	MT135043	MG011340	KT029139	KF600651	KF923911	MN988668	

3. FEATURE LIST TO CLASSIFY DIFFERENT CORONAVIRUSES

Table S2

TACAACATACCCTACTAATGT	CACCACTATTTGGGTTAACCC
TAGCATTAACCATGATTTGTT	GTTGAATGATCTTTGCTTCTC
GGTGATAAATTAGATCAGTTC	AGCGATAGGTTTTATCGACTT
CTAAAGCATACAATGTAACAC	CAAAAGTAACTAGTGCTATGC
TACCAGTGATTTAGGTAGTCT	TGCTTTAGATCAAGCTATTTC
AGGTCAAGAGCAAACTAACTG	TAACACTTACCTGGAGGGTTC
GAAGAGCTACCAGACGAATTC	GATTCTCAAATTTCTCATTAC
TGAATATAAATGATTGCACTC	CGGCTTGATTCAATTCAAGCC
GAAGGTAGTCGTGTGCCACAC	TATTAACAAGGACACTTATGC
TGTGGAGTATCCCATCATTTC	TGGGCTCTTGCCAACCAATTC
CTTCGGGAACGTGGTTGACCT	TATTGACTATAGACACTATTC
TGACTGCTAACAATCTAACTG	ATGCTATGGGCAAACCTGTGC
CTTGGTTCACCGCTCTCACTC	TATTGCTGACCTTGCTTGTGC
CAAAATCAGCGAAATGCACCC	TGACAGCTAACAATCTAACTG
GATTAATGATATGGTTTATTC	CAAAGTCTACTATGGTAATGC
TGTGTTTAGTCAAGTTGATTT	CAATGTTTATTGTTATAACAC
TGCTAACAATCTAACTGCACC	AAGAGAGAAGTTGCTTCATTT
GCTGAATAAGCATATTGACGC	TAAAGTTCATTTTTATTACCT
GGGTTGCAACTGAGGGAGCCT	TGGCTGCAACGTAACTGACGT
AATTGGAATGTGGAGTATCCC	CTGTGGCTACCTTGACGGCGC
CTTGACAGATTGAACCAGCTT	CAAATAGCACCTGTTCCAGCT
AGGCGGCAGTCAAGCCTCTTC	AATGTCTAAACTAAACGATGT
TATTTGCAATTCAGCTGTTGC	TGCTGATGATTCAGGTACTCC
GGGCCAGAAGCTGGACTTCCC	GACCTTAAATTCAGACAACGT
AGACGTGGTCCAGAACAAACC	CATGCTGTACCGACTCTCTTT
TATACTCAACTGTGTCAATAC	TACAGAATTTTAAGGAAACGC
TGGCCGCAAATTGCACAATTT	

4. ORIGINAL FORMAT OF PCR RESULTS

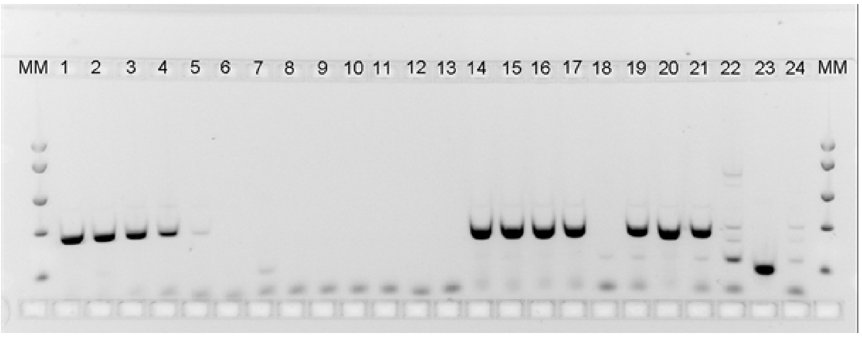

Fig. S8. Original format of PCR Results.

5. PROPOSED PIPELINE FOR AUTOMATED PRIMER DESIGN

The main paper *Classification and Specific Primer Design for Accurate Detection of SARS-CoV-2 Using Deep Learning* presented a series of experiments with the aim to explore the possibility of automatic primer design using artificial intelligence. Once this possibility is validated, the pipeline we propose to automatically design primers for a new virus will be:

Collect samples of the new virus, along with samples of other virus of the same family (the methodology showed promising results with just 500 samples total, but the more, the better);

Train a CNN classifier in a cross-validation on the dataset built in the previous step. If the average CNN classification exceeds a given threshold for a quality metric (e.g. classification accuracy above 95%), this is a good indication that the CNN learned meaningful features, and the pipline can proceed to the following step;

Extract features using the max-pooling of the CNN filters, extract the position in the original data of 21-bps features;

Prune features from the feature set obtained at the previous step, by first discarding those that cannot qualify as primers due to their structure (e.g. they contain missing bps "N"), and then performing a feature selection on the remaining ones. If, after this step, it's possible to identify a small number of features that still provide a high classification accuracy, it is possible to proceed to the next step;

Test features as primers using an in-silico assessment of their quality, e.g. Primer3Plus. If the feature is considered as an "acceptable" left primer, the software will generate a right primer for the base sequence;

In-vitro primer testing performed in a lab. If the primers are confirmed to be qualitatively satisfying, the process is complete and the primers can be used on the market.
